# No Hot Spot Mutations *CHRNE *c.1327 delG, *CHAT* c.914T>C, and *RAPSN* c.264C>A in Iranian Patients with Congenital Myasthenic Syndrome

**Published:** 2019

**Authors:** Sima PARVIZI OMRAN, Massod HOUSHMAND, Donkor DOMINIC, Zahra FARJAMI, Parvaneh KARIMZADEH

**Affiliations:** 1 Department of Biology, Damghan Branch, Islamic Azad University, Damghan, Iran; 2 Department of Human Genetics, National Institute of Genetic Engineerin -Biotechnology, Tehran, Iran; 3 Department of Biology, Concordia University, Montreal, Canada.; 4 Department of Modern Sciences and Technologies, Faculty of Medicine, Mashhad University of Medical Sciences; 5 Pediatric Neurology Research Center, Research Institute for Children’s Health, Shahid Beheshti University of Medical Sciences, Tehran, Iran; 6 6.Pediatric Neurology Department, Mofid Children’s Hospital, Faculty of Medicine, Shahid Beheshti University of Medical Sciences, Tehran, Iran

**Keywords:** Congenital myasthenic syndrome, CHRNE, CHAT, Rapsyn, Hot spot mutation

## Abstract

**Objectives:**

We aimed to perform genetic testing and clinical data of patients with Congenital Myasthenic Syndrome, a rare disorder caused by mutations in genes encoding molecules expressed in the neuromuscular junction and constitutes fatigable muscle weakness.

**Materials & Methods:**

Sixteen patients were screened in Taban Clinic, Tehran, Iran from 2014 to 2015 for the hot spot mutations in known CMSs genes (*CHRNE, CHAT*, *RAPSN*) based on clinical data. PCR was performed and then direct DNA sequencing was done for mutation identification.

**Results:**

Most patients represented the criteria of Congenital Myasthenic Syndrome in view of early ptosis, motor delay, normal mental development, easy fatigability, decrement in repetitive nerve stimulation test of EMG-NCV and a negative result for antibody against of acetylcholine receptor. No variations were found in the mutational analysis of the *CHRNE *gene. Analysis of *CHAT *gene revealed c.358G>A (P. A120T) variation in 9 patients. In the gene *RAPSN,* polymorphism c.456T>C )P.Y152Y) and polymorphism c.193-15C>T (IVS1-15C>T) were identified in 11 and one patients, respectively.

**Conclusion:**

The common founder mutations of involved genes in CMSs could be very rare among ethnic Iranian. Screening of the entire genes would be efficient to distinguish the specific mutations in specific ethnicity.

## Introduction

Congenital Myasthenic syndromes (CMSs) are considered as an extremely rare heterogeneous group of disorders that caused dysfunction of neuromuscular transmission. In CMSs, impaired neural transmission has identified in pre-synaptic, synaptic and most frequently in post-synaptic stages. Mainly, post-synaptic CMSs are caused by deficiency or kinetic abnormalities of acetylcholine receptor (AChR) and are much more frequent than pre-synaptic or synaptic form. The cardinal presentations of CMSs are hypotonia, episodic apnea, ptosis and fatigability that appear in early infancy or childhood. Patients have a significant variability in the clinical phenotype, onset, course of the disease, and response to treatment (1, 2).

Accurate molecular mechanisms arising from the genetic defect help confirmation of the diagnosis and also determine a proper therapy (3, 4). 

To date, 25 different genes encoding proteins involved in neuromuscular junction have been identified in association with CMSs. Mutations in the epsilon subunit of the nicotinic acetylcholine receptor determined the commonest cause but the *RAPSN* gene was more likely to be the second causative mutant gene for the disease. *RAPNS *gene plays a pivotal role in AChR clustering in the post-synaptic membrane of the neuromuscular junction (2). The missense c.264C>A, p. Asn88Lys mutation in the *RAPSN* is the fundamental defect reported particularly among European (1).

On the other hand, a possible mutation in *CHAT *which is first responsible gene for presynaptic CMS has been proposed to be scrutinized according to the literature.* CHAT* encodes the enzyme choline acetyltransferase and its deficiency is known for causing CMS with apnoeic episodes (EA) (5).

The objectives of our study tackled the screening for founder mutations involving in *CHRNE, CHAT,* and *RAPSN*. It has also compared the frequency of founder mutation p. Asn88Lys in *RAPSN* in different populations.

## Materials & Methods

The study cases consisted of 16 individuals (10 males and 6 females) referred from the Department of Pediatric Neurology, Mofid Children’s Hospital Tehran, Iran from 2014 to 2015. The diagnosis of CMS was performed on the basis of the clinical features, the absence of anti-AChR antibodies, electromyography examination including; single fiber test and the positive familiar history (3).

**Figure 1:**
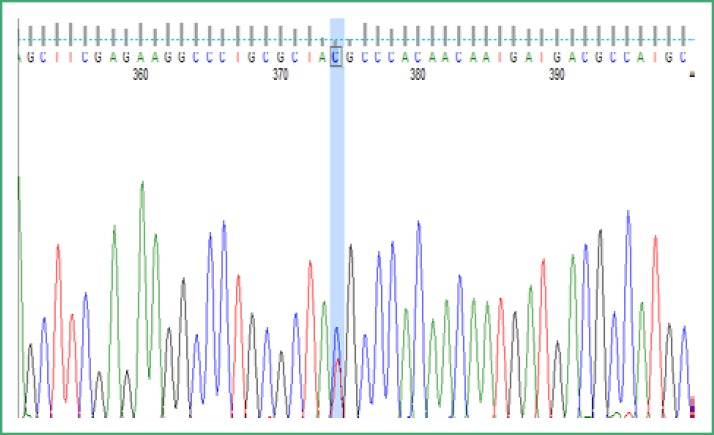
Sequence electropherogram showing a polymorphism c.456T>C *RAPSN* in heterozygous state

**Table 1:** Clinical presentation of the affected individuals

**Patient ID**	**Age at onset, sex**	**Consanguineous** **Marriage**	**Presentation**	**Antibody Status:** **Anti-AChR**	**Repetitive N. stimulation**	**Treatment/Response to Treatment**
Case 1	2 yr, M	Positive	Ptosis, easy fatigability, that was worst with activity and lack of sleep	Negative	At first was negative	Good response to Prozac and Salbutamol
Case 2	18months, M	Positive	Ptosis, easy fatigability, motor delay	Negative	No EMG-NCV	No treatment
Case 3	M 10yr	Negative	Ptosis, easy fatigability	Negative	No EMG-NCV	No treatment
Case 4	M,14months	Positive	Ptosis, easy fatigability	Negative	No EMG-NCV	No treatment
Case 5	1.5 yr	Positive	Ptosis, Ataxia, Hypotonia and normal mental development	Negative	Negative	No response to Salbutamol, Pseudoephedrine and mestinone, Partial response to Prozac
Case 6	8.5months, F	Negative	Ptosis, easy fatigability	Negative	No EMG-NCV	No treatment
Case 7	17 months, F	Positive	Ptosis, easy fatigability	Negative	No EMG-NCV	No treatment
Case 8	4 yr	Negative	Ptosis, easy fatigability, that was worst with activity and lack of sleep	Negative	Positive decrement	No response to Salbutamol, Pseudoephedrine and mestinone, Partial response to Prozac
Case 9	10 months, M	Positive	Ptosis, hypotonia, Tracheomalacia, Respiratory Distress	Negative	No EMG-NCV	Good response to Pseudoephedrine and Prozac
Case 10	2.5months, M	Positive	Ptosis, hypotonia that was worst with lack of sleep	Negative	Negative in 4 months old	Good response to Prozac
Case 11	2 yr, M	Positive	Ptosis, easy fatigability, that was worst with activity and lack of sleep	Negative	Positive decrement	Good response to Prozac
Case 12	7 yr, M	Positive	Ptosis, Swallowing disorder, Proximal weakness, easy fatigability	Negative	Positive decrement	No treatment
Case 13	20months, F	positive	Hypotonia, Respiratory distress, Pneumonia	Negative	No EMG-NCV	No response to Prydostigmine
Case 14	8months, M	Positive	Ptosis, easy fatigability	Negative	No EMG-NCV	No treatment
Case 15	2yr, M	Positive	Ptosis, easy fatigability	Negative	No EMG-NCV	No treatment
Case 16	8 yr	Positive	Motor delay	Negative	Positive Decrement	Good Response to Pseudoephedrine

**Table 2:** PCR Primes sequences and amplicon size of candidate genes

Primer	Sequence	Exon Amplicon Size (bp)	TM (C)
*CHRNE *- Forward prime *CHRNE *- Reverse primer	5´-GAGCGAGCTCGTGTTTGAG -3 5´-GAGACAGTGGTGGGCCTCT -3´	11 300	63 °C
*CHRNE *– Forward primer *CHRNE *– Reverse primer	5´-CTGGCTCCTGCAGCTGCCTC-3´ 5´-CTGGAGATGGGTGGGAAATTG-3´	12 238	**61 **°C
*CHAT*- Forward primer *CHAT*- Reverse primer	5´-GAGGTGGAGGGTTTGTGACAGG-3´ 5´CTAGAAGCAAGGGCATGTAGGTG-3´	5 231	62 °C
*RAPSN- *Forward primer *RAPSN*- Reverse primer	5´-CTTTGGGATCTGCTGCTTTGGGT-3´ 5´- AAGGAGGGCTGAATGAGGTAGTGC-3´	2 570	58.1 °C

Note: Abbreviations: TM, temperature; PCR, polymerase chain reaction; bp: base pair;

**Table 3:** Frequency of (c.264C>A; p. Asn88Lys. *RAPSN*) in different population

Ethnic Origin, Reference	number of patients with N88K Mutation /Number of patients with CMS	Mutation Positive (%)
European and Indian (11)	21/21	100
European, Asian (15)	16/16	100
Asian, Iranian Jewish, European (16)	18/37	48
French (17)	5/20	25
Around the world (2)	31/39	11
Western European (9)	12/120	10
German, Italian, Spanish, Switzerland: Swiss, Czech, Canadian (6)	39/680	5
Brazilian (18)	0/25	0
Japanese (19)	0/6	0
Iranian	0/16	0

In neurological examination, the patients had motor delay, Ptosis and muscle weakness that was worst with activity and lack of sleep. Deep tendon reflexes were diminished and plantar reflexes were downward. We evaluated all patients with electromyography examination and the result showed more than 10% decrement that confirmed the diagnosis of Myasthenia. Furthermore, antibodies against the Ach choline receptors were negative; therefore, they are not the cases of Myasthenia gravis. 82% of our patients were offspring of consanguineous marriage and the sign and symptoms were started in early weeks and months of life. On the other hand, the other diagnoses about motor delay and lower motor neuron diseases such as mitochondrial disorders, and congenital myopathy hypothyroidism were ruled out.

In genetic study, blood samples were obtained from the patients and genomic DNA was isolated using blood DNA extraction kit (MBST, Tehran, Iran). Target mutation analysis for previously reported mutations in 11 and 12 of *CHRNE, *exon 5 of *CHAT *and, exon 2 of *RAPSN *gene was performed with primers in Table 1. In addition, Primer sequence for exon 2 of *RAPSN* gene was ordered (6).

 The PCR reactions were performed in a thermal cycler (Techne, Genius, UK) for 5 min at 95 °C followed by 35 cycles of denaturation for 1 min at 94 °C, annealing for 1 min depending on melting temperature of primers (Table 2), primer extension for 30 sec at 72 °C with final 5 min extension at 72 °C. The amplified PCR products were sequenced by ABI3100 system. Finch TV software was used to align and analyze the DNA sequences and compared with gene bank.

## Results

**We screened in all patients for the common mutations** ***CHRNE*** **p. Glu443LysfsTer64** **and *CHAT* p. lle305Thr.Subsequently, we sequenced *RAPSN *gene for the known mutations p. Asn88Lys, p. Arg91Cys, p. Glu94Lys, p. Arg164Cys, and p. Val165Met widely reported. Although patient’ clinical phenotype is compatible with CMS, no pathogenic mutations were found by sequencing *CHRNE*,* CHAT*, and *RAPSN*. **

Of the 16 patients recruited, nine patients identified with variation c.3558G>A (p. A120T) in *CHAT*. In the gene *RAPSN* two previously published polymorphisms were detected: c.456T>C )P.Y152Y) and c.193-15C>T (IVS1-15C>T) (3). Precisely, among 13 patients with polymorphism c.456T>C; 11 were homozygous and 2 were heterozygous. Only one patient harbored the frequent polymorphism c.193-15C>T (IVS1-15C>T) in the homozygous state. As an illustration, Figure 1 showed the polymorphism c.456T>C *RAPSN *in heterozygous state.

## Discussion

Screening of hotspot mutations in *CHRNE* and *CHAT* and *RAPSN *have been extensively reported by many studies. Precisely, Targeted assessment were included mutations (c.1327 delG; exon 12) (7) in *CHRNE*, (c.914T>C; exon 5) (8) in *CHAT* and (c.264C>A, exon 2) (9) in *RAPSN*. 

The *CHRNE* mutation c.1327delG were found in up to 50% of individuals of European Roma (7, 10). Whereas the *RAPSN* c.264C>A mutation due to a founder effect accounting for about 90% of patients’ origination from Europe (11). Table 3 reveals the distribution of mutation c.264C>A, p. Asn88Lys in *RAPSN* across geographical regions.

Besides, the vast majority of variations in *CHRNE *and *RAPSN* genes were dispersed over the entire gene. To date, in the Mayo clinic cohort of CMS patients of mostly European origin, *CHRNE* and *RAPSN* mutations were found 51% and 14%, respectively (6). Different frequencies were found in Israel cohort, where 20% had mutations in *CHREN*, 37% in *RAPSN,* and 3% in *CHAT *(12). The mutation c.-38A>G *RAPSN* occurs in a cohort of Iranian and/or Iraqi Jewish origin patients (13).

No pathogenic mutations were detected in our candidate gene approach. The variations were detected in *CHRNE* and *CHAT *genes did not show any amino acids exchanges. The exonic SNP 456T>C,rs7111873 in *RAPSN *does not underlie amino acid substitution (3, 11). The intronic SNP IVS1-15C>T may result from transcriptional or conformational protein changes (14), but this would need more investigation. Therefore, none of the known polymorphisms were found to be significantly related to CMSs.As accordance with the high rate of hotspot mutations c.1327delG, c.264C>A of *CHRNE* and *RAPSN*, respectively, in European descent and also our similarities to European in terms of ethnicity, we postulated that the mutations will be observed at least 5% in our series. 

The frequencies of SNPs rs7111873 of *RAPSN *and rs3810950 in *CHAT* which found in our cohort differ from the SNP frequencies reported in Iranome (http://iranome.com/) including 0.7175 in rs711873 of *RAPSN* and 0.2594 of *CHAT*. There are two possible reasons for differentiation: First, CMS is a rare condition; larger sample size may facilitate finding the known European founder mutation in our population (9). Second, Iran is one of the most multi-ethnic states. It consists of different ethnicities including Lure, Turk, Mazani, Fars, and Kurd and other subpopulations. Thus, to obtain ethnic-specific mutation for CMSs expanded genetic tests and centralized clinical management would help to advance research opportunities.

A negative screening for the hotspot mutations does not rule out the possibility of underlying the mutations of the genes. Further studies that screen *CHRNE, CHAT*, and *RAPSN* genes full sequence are warranted to throw light and elucidate the mutational basis of CMSs in Iranian. The use of next-generation sequencing (NGS) method in spite of being expensive (12), may help to identify the mutated genes. 

**In conclusion, **the outcome of this study has extended the genotype spectrum of Iranian with CMS, conferring a way to a more effective method for career detection, genetic diagnosis and counseling of Iranian patients with CMS disorders. 
